# Study on the expression of the rate constant *α* of the creep equation by modified θ projection applied to turbine materials

**DOI:** 10.1016/j.heliyon.2019.e02618

**Published:** 2019-10-19

**Authors:** Hideo Hiraguchi

**Affiliations:** The Institution of Professional Engineers, Japan (IPEJ), 5-8 3-Chome Shibakoen, Minato-ku, Tokyo 105-0011, Japan

**Keywords:** Materials science, Computational mathematics, Discrete cosine transform, Creep equation, Interpolation, Rate constant α, Modified θ projection

## Abstract

It is well-known that the creep equation obtained by the modified θ projection [2] describes well from the primary creep region to the tertiary creep region. However, unlike the power law such as that applied by the Bailey-Norton method, the stress variables and temperature variables are not found in the equation coefficients. Therefore, the users of this equation must find functions containing temperature variables and stress variables to display the equation coefficients. Thus, among the three coefficients A, α, and B included in the equation of the modified θ projection, the rate constant α, which exerts the largest influence on the curvature and the minimum creep strain rate of the creep curve [3], was selected as the object of investigation. Moreover, by considering the Cr-Mo-V steel and the Ni-based superalloy as examples, the expression of α was investigated. As a result, it was found that the discrete cosine transform and series can be applied not only to the coefficients of the creep equation but also to the creep equation itself. It is very important that the Fourier transform, which is considered to be applicable only to periodic functions, can be applied to non-periodic functions like a creep equation or its coefficients without apodizing such as windowing [9].

## 1 Introduction

The modified θ projection, which is one of the creep equations, is often used to predict the creep damage rate at each temperature and pressure of gas turbine materials by using interpolation and extrapolation. The modified θ projection, which is a modified one of the Evans and Wilshire equation [1], is expressed by Eq. (1), and consists of the three coefficients *A*, *α*, and *B* [2]**.**

(1) *ε* = *ε*_0_ + *A*{1-exp(-*αt*)} + *B*{exp(*αt*)-1}

where *ε* is the total strain, *ε*_0_ is the initial strain, and *t* is the creep test time.

Coefficients *A*, *α*, and *B* have been found to depend on stress and temperature [2], respectively. In this study, we focused on the rate constant *α*, which exerts the largest influence, among these three coefficients, on both the curvature and the minimum creep strain rate of the creep curve. The results obtained by investigating the expression of the rate constant *α* in Eq. (1) are presented in this paper. Generally, *α* is represented by the power of stress *σ*, as expressed by Eq. (2) [3].

**Fig. 1:**
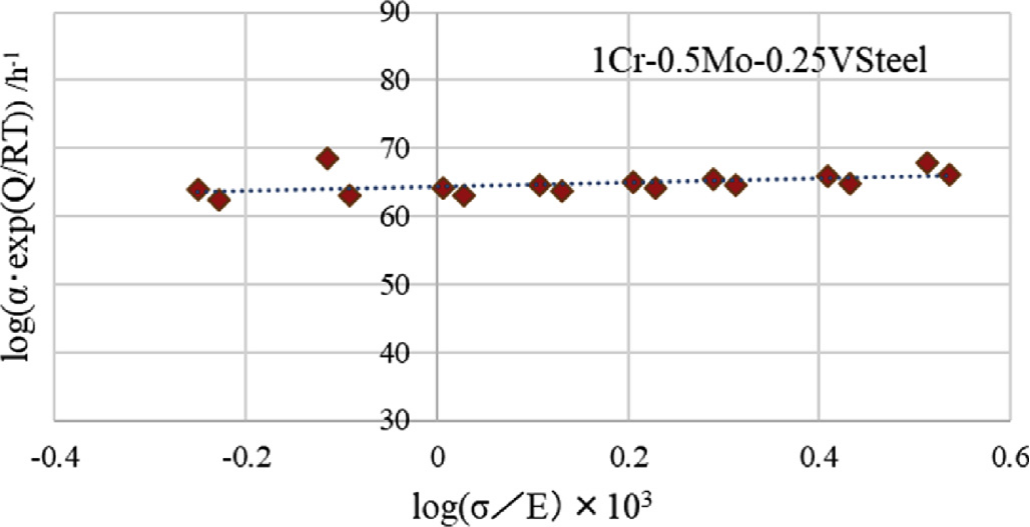
Relationship between rate constant α compensated by temperature and stress normalized by Young's modulus.

(2) *α* = *N*·(*σ*/*E*)^*n*^·exp(-*Qc*/*RT*)

where *σ* represents stress, *E* is Young's modulus, *Qc* is the activation energy of the creep strain rate, *R* is a gas constant, and *T* represents temperature. As shown in Fig. 1, the interpolation and extrapolation are typically performed using the linear relationship between the natural log_e_ (*α*) and log_e_(σ/E). However, as described later in Fig. 3, the values of *α* obtained by interpolation from the linear relationship in the high stress range are plotted far away from the measured values, in comparison with other expected values that can be calculated without using the natural logarithm.

So far, in order to apply the nonlinear least square method to the measured values, it was necessary to find a function similar to the curve drawn with the measured values and minimize the sum of the squares of the residuals. However, in this method, depending on the type of curve drawn with the measured values, a function that draws a similar curve may not be found. In this case, the nonlinear least square method will not be able to be implemented. Further, the nonlinear least square method has a problem that the theoretical curve does not pass through all the measured values.

Therefore, in this paper, we present the various expressions for *α* calculated without using the natural logarithm to achieve a more accurate interpolation prediction. In addition, among these expressions, because we found out that the discrete cosine transform and series can pass through all the measured values and the calculated values by interpolation are reasonable, we also report this result.

The content of this research is a modified version of what was presented at Asian Congress on Gas Turbines 2018 [8]. In the presentation, the values of a few rate constant α calculated from two-dimensional discrete cosine transform indicated negative values. In later research, we have discovered a new way to extinguish those negative values. Therefore we report this new way in this paper.

## 2 Experimental

### 2.1 Computer experimental procedures

As the data used for the calculation of the rate constant *α*, the 1Cr-0.5Mo-0.25V turbine bolt materials and Ni-based 16Cr-8.5Co-3.5Al-3.5Ti-2.6W-1.8Mo-0.9Nb superalloy gas turbine blade materials were selected from the creep sheets (No.44, No.49A) of the database of the National Institute for Materials Science (NIMS).

From the abovementioned data, the rate constant *α* was obtained using the least squares method under each stress of 137, 157, 177, 196, 216, 235, 265, and 294 MPa at 500 °C, and each stress of 69, 78, 98, 118, 137, 157, 177, 196, 216, 235, 265, and 294 MPa at 550 °C for 1Cr-0.5Mo-0.25V steel (No.44).

Similarly, *α* was obtained under each stress of 300, 350, 400, 500, 550, and 600 MPa at 750 °C, each stress of 200, 250, 350, 400, and 500 MPa at 800 °C, each stress of 100, 150, 200, 300, and 400 MPa at 850 °C, and each stress of 100, 150, 200, 250, and 300 MPa at 900 °C for the Ni-based superalloy (No.49A).

**Fig. 2:**
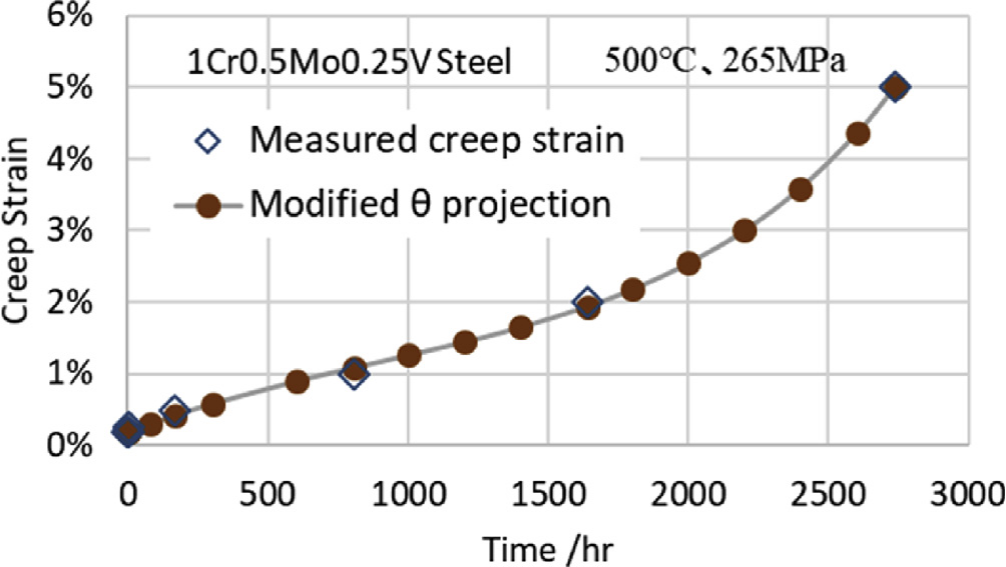
Creep curve using modified θ projection.

For example, Fig. 2 shows the fitting curve obtained by the modified θ projection of the 1CrMoV steel at 500 °C and 265 MPa. In Fig. 2, when the measurement time is 170 h, the measured value is 0.5%, and the calculated value is 0.42%. When the measurement time is 805 h, the measured value is 1.00% and the calculated value is 1.08%. When the measurement time is 1640 h, the measured value is 2.00%, and the calculated value is 1.94%. Thus, the creep curve of the modified θ projection fits the measured values well. However it does not pass through all the measured points.

## 3 Theory/calculation

### 3.1 N-spline function technique

The spline function is a low-order polynomial in each small section. It was introduced for smoothing the statistical data by Schoenberg and it is a piecewise polynomial connected smoothly in its entirety. In this computer experiment, the smoothest N-Spline function was selected. If n+1 interpolation points (*x*_0_, *y*_0_), (*x*_1_, *y*_1_), … (*x*_*n*_, *y*_*n*_) are given and the i^th^ interval [*X*_*i*_, *Y*_*i*_] is expressed as follows [4]:

(3) *P*_*i*_*(x)* = *C*_1*,i*_ + *C*_2*,i*_ * (*x-x*_*i-*1_) + *C*_3*,i*_ * (*x-x*_*i-*1_) ^2^ + *C*_4*,i*_ * (*x-x*_*i-*1_)^3^

where, *C*_*j, i*_ (*j* = 1, 2, 3, 4) is a coefficient of the third-order equation.

**Fig. 3:**
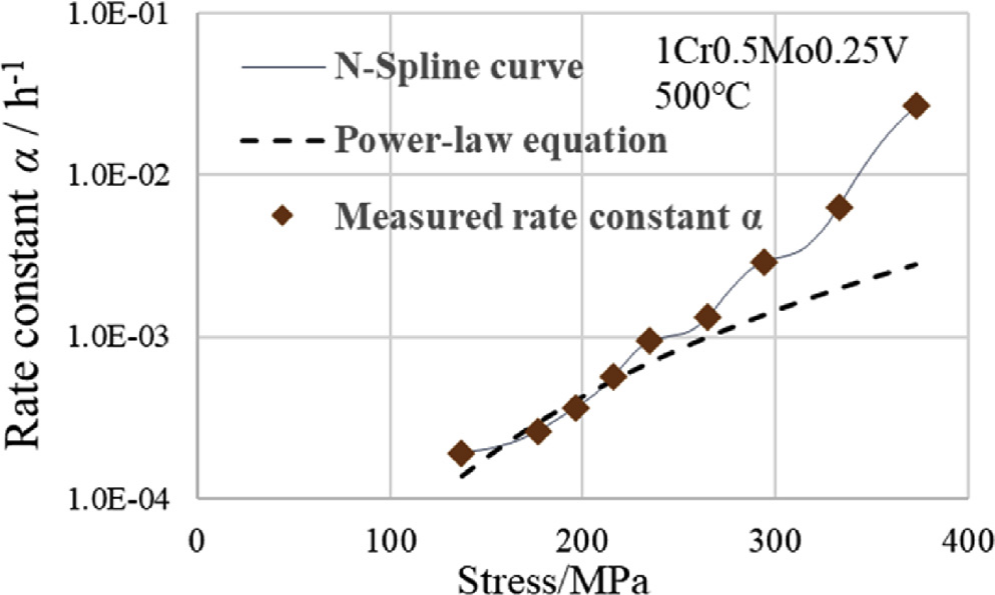
Relation between rate constant α and stress.

Fig. 3 indicates the curve of the power-law equation (Eq. (2)) by the dashed lines at 500 °C for the 1CrMoV steel. In Fig. 3, it can be seen that the three points at the right end of the high stress side are far away from the dashed line.

In this situation, because it is not possible to accurately predict the rate constant *α* for 265 MPa or more, it is therefore not possible to increase the accuracy of the creep strain estimation by using the modified θ projection with this value of *α*. Therefore, an approximation was attempted using the N-Spline function from Eq. (3). The result is indicated by the solid line in Fig. 3, where it can be seen that the prediction by interpolation was more accurate, because the N-Spline curve smoothly passes through all of the measured points.

### 3.2 Modified θ projection equation technique

**Fig. 4:**
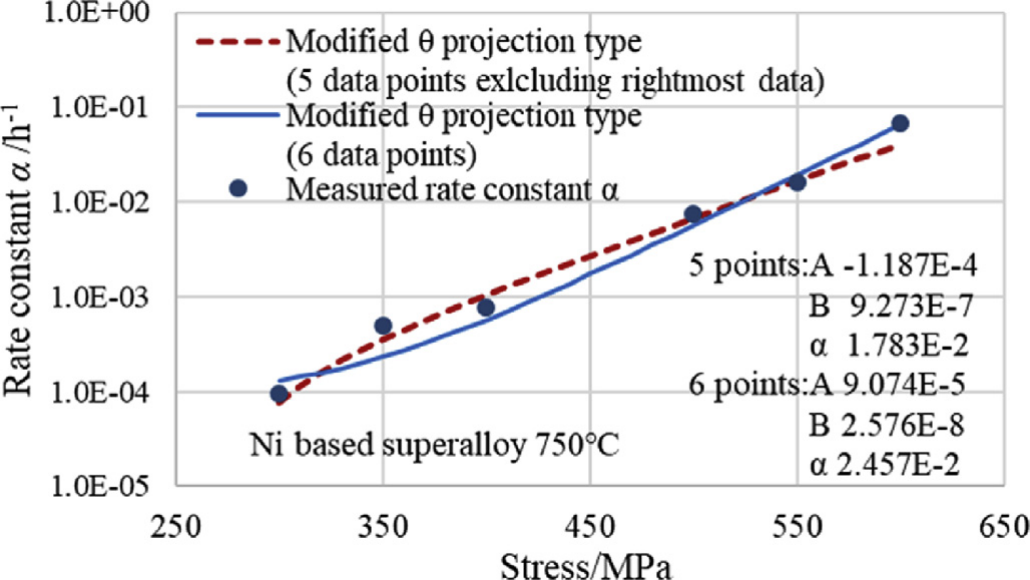
Comparison of modified θ projection type and measured α versus stress curves at 750 °C of Ni based alloy.

Fig. 3 shows that the shape of the relationship curve between the measured rate constant *α* and stress is similar to that of the relationship curve between the creep strain and time. Therefore, we attempted to apply the equation of the modified θ projection, which is typically used to obtain the relationship curve between the creep strain and time, to fit the relationship curve between the rate constant *α* and stress. Fig. 4 shows an approximate curve using Eq. (4) of the modified θ projection type. This curve demonstrates the relationship between the rate constant *α* and stress *σ* at 750 °C for the Ni-based alloy newer than 1Cr-0.5Mo-0.25V steel. When *α* was displayed on the vertical axis in Fig. 4, the vertical axis was displayed as a common logarithmic axis.

(4) *α* = *A*1{1-exp(-*C*1·*σ*)} + *B*1{exp(*C*1·*σ*)-1}

Fig. 4 shows that the modified θ projection type curve fits the measured values very well. The modified θ projection type calculated with five points of data, excluding one point of data at the right end, is a better fit. However, the values of *α* are negative at 150 MPa or less. Moreover, in the modified θ projection type calculated with six points of data including one point of data at the right end, the estimated value for *α* is slightly further away from the value measured at 350 MPa. However, because the estimated *α* value takes a positive value down to 0 MPa, it is suitable for extrapolation to the low stress side. Therefore, when extrapolating to a low stress region, it is better to use the modified θ projection type calculated with six points of data in this data set. When interpolating between the measurement points from 300 MPa to 550 MPa, it is better to use the modified θ projection type calculated with five points of data. By including or excluding one point of the rightmost high stress, the modified θ projection type can respond more flexibly than the power law, regardless of whether the important point is on the high stress side, medium stress side, or low stress side.

**Fig. 5:**
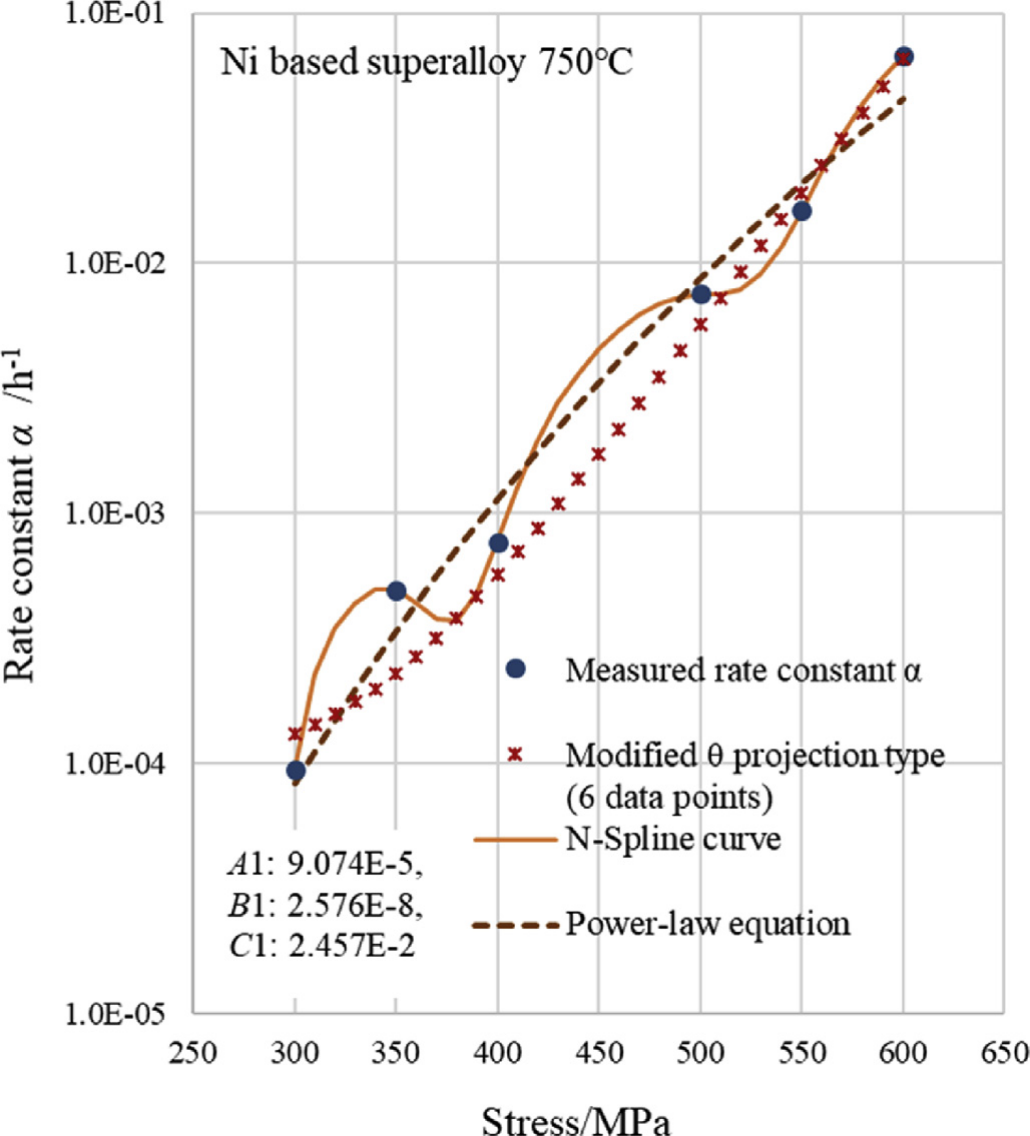
Comparison of three types of equations.

Fig. 5 shows the following three types of curves: the modified θ projection type (Eq. (4)), N-Spline function (Eq. (3)), and power-law equation (Eq. (2)). Fig. 5 shows that the modified θ projection type fits the measured value better than the power-law equation at 400 MPa or at a higher stress region, except for 500 MPa, and particularly at 550 MPa and 600 MPa. The N-Spline curve passes through all of the measured points, but the N-Spline curve between 400 MPa and 500 MPa bulges in comparison with the other two equations. Conversely, the N-Spline curve between 500 MPa and 550 MPa bulges less in comparison with the other two equations. Because the rate constant *α* is slightly larger between 400 MPa and 500 MPa, the creep strain is assumed to be on the safe side. Additionally, because the rate constant *α* is slightly smaller between 500 MPa and 550 MPa, the creep strain is assumed to be on the dangerous side. Between 550 MPa and 600 MPa, the modified θ projection and the N-Spline function fit the measured values approximately to the same extent. Fig. 5 shows that the N-Spline curve between 300 MPa and 400 MPa passes all of the measured points. However, the interpolation curve of the N-Spline resembles a sine curve. Therefore, in this range, the N-Spline curve cannot be used to estimate by interpolation. Moreover, the power-law equation fits the measured value better than the modified θ projection curve. From the above considerations, it can be said that the N-Spline function is suitable if the calculated value must always be consistent with each measured value, in addition to requiring good estimation accuracy in the high stress regions of 550 MPa and 600 MPa. However, if the calculated value does not need to be precisely consistent with each measured value, but requires good estimation accuracy in the region of 400 MPa or more, the modified θ projection type is suitable because the values of *α* according to the modified θ projection type are the closest ones to the value measured at 400 MPa, 550 MPa, and 600 MPa. Additionally, they are as close to the measured value at 500 MPa as they are to the value obtained by the power-law equation. The power-law equation is suitable in the region of 400 MPa or less. Because the measured values are non-linear, and in the case of paying attention to the higher pressure side, as shown in Fig. 5, we considered that the significance of using the modified θ projection is large.

**Fig. 6:**
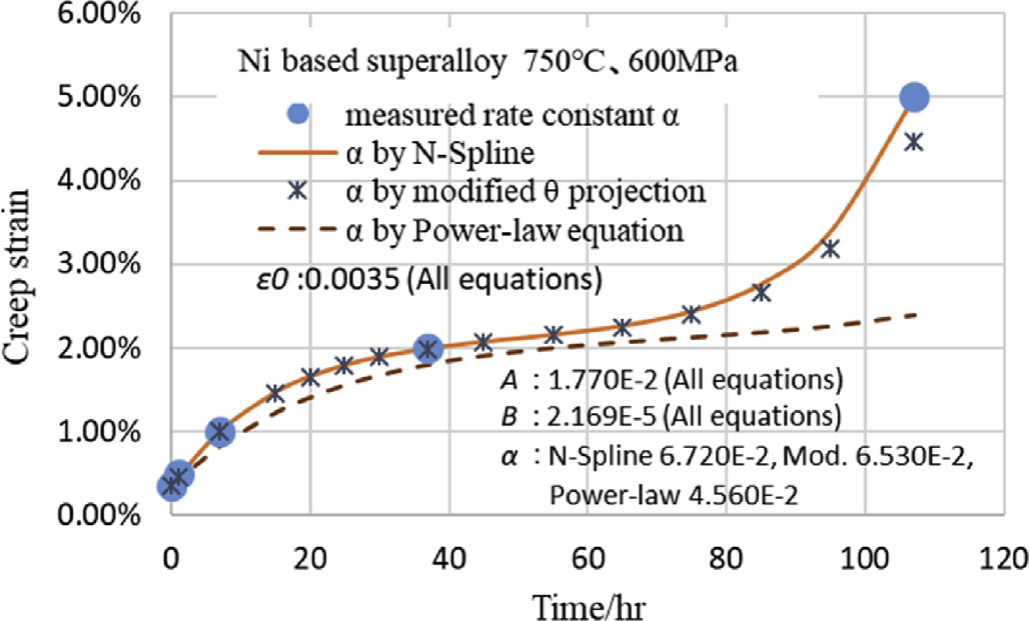
Creep strain curves by modified θ projection using α from various equations.

As an example, Fig. 6 shows the confirmation result with regard to how much the difference in the value of *α* estimated by the above three equations appears as a difference in the creep strain curves with the creep data of the Ni-based alloy at 750 °C and 600 MPa. The creep strain after 100 h was 3.9% using the *α* obtained by the N-Spline, 3.6% using the *α* obtained by the modified θ projection type, and 2.3% using the *α* obtained by the power-law equation. Therefore, when *α* is estimated by interpolation, the creep strain greatly changes depending on which equation is used.

### 3.3 Two-dimensional representation of rate constant *α* by temperature and stress using modified θ projection type

In the previous section, the relationship between *α* and stress was successfully expressed by the modified θ projection type. Therefore, in this section, the relationship between *α* and temperature *T* is expressed by Eq. (5) of the modified θ projection type.

(5) *α* = *A*2{1-exp(-*C*2·*T*)} + *B*2{exp(*C*2·*T*)-1}

**Fig. 7:**
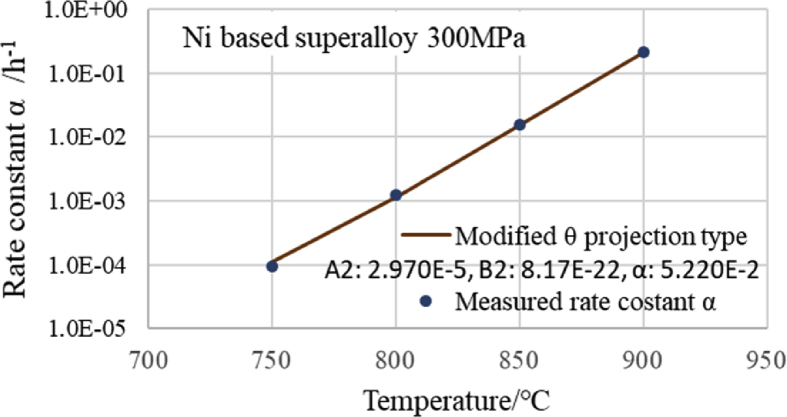
Relationship between α and temperature.

The results are presented in Fig. 7, where it can be seen that the value of *α*, which was obtained in each stress by the modified θ projection type, fits the measured value adequately. Because Eq. (5) has a first term and a second term, it is capable of flexibly responding to linear and non-linear situations.

**Fig. 8:**
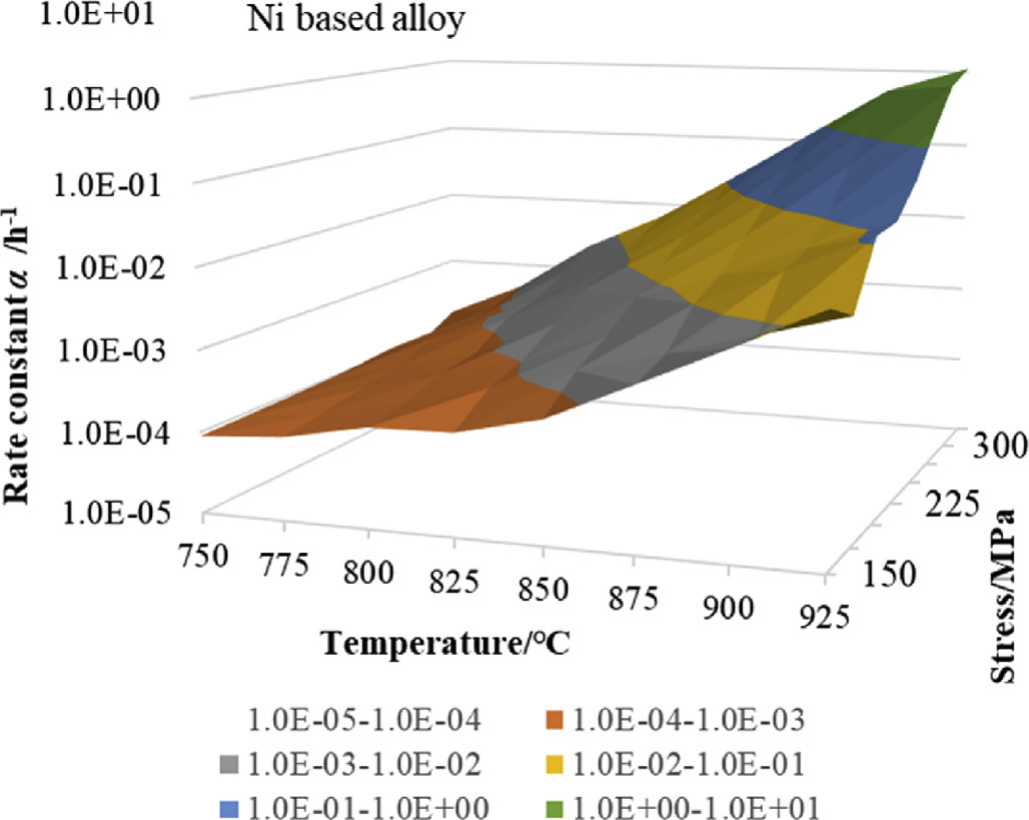
3D contour map of rate constant α by stress and temperature.

Therefore, a three-dimensional (3D) contour diagram of *α* was drawn by considering the stress as the X-axis, the temperature as the Y-axis, and the rate constant *α* as the Z-axis. This made it possible to grasp the relationship between *α* and the 2D variable (*σ*, *T*) from one 3D graph. First, eight pieces of stress data, namely, 150, 175, 200, 225, 250, 275, 300, and 325 MPa, were selected as the stress data on the X-axis, and eight pieces of temperature data, namely, 750, 775, 800, 825, 850, 875, 900, and 925 °C, were selected as the temperature data on the Y-axis. Subsequently, the rate constant *α*_*ij*_ for each 8 × 8 (*σ*_*ij*_, *T*_*ij*_) (*i* = 0 to 7, *j* = 0 to 7) data was calculated. Specifically, the coefficients *A*1, *B*1, and *C*1 of Eq. (4) were calculated at each temperature from the creep data of the Ni-based alloys at 750, 800, 850, and 900 °C in the NIMS database. From these coefficients, *α* was calculated for stresses that are not found in the NIMS database. Next, the coefficients *A*2, *B*2, and *C*2 of Eq. (5) were calculated for each stress that was equally spaced at 25 MPa, namely, 150, 175, 200, 225, 250, 275, 300, and 325 MPa, by using the previously calculated value of *α*. By adding the value of *α* that was estimated at the temperatures of 775, 825, 875, and 925 °C to the abovementioned temperature data, the 8×8 *α*_*ij*_ (*i* = 0 to 7, *j* = 0 to 7) were calculated by Eqs. (4) and (5). Fig. 8 shows a 3D contour map for the previously obtained value of *α*. However, the contour curves are not the curves of the modified θ projection, but rather those of the linear interpolation. As shown in Fig. 8, the value of *α* increased as the temperature and stress increased.

### 3.4 Discrete cosine transform technique

#### 3.4.1 Application to creep curve

The relationship between the creep strain and time obtained by the modified θ projection in Eq. (1) is known to provide a fairly good approximation from the primary creep region to the tertiary creep region. However, it does not necessarily pass the measured points, unlike the N-Spline function. Moreover, the N-Spline curve has drawbacks such as a tendency to draw a sine curve between two points. Therefore, a discrete cosine transform function is proposed, which always passes through the measured points like the N-Spline and provides a good approximation from the primary creep region to the tertiary creep region similar to the modified θ projection. The discrete cosine transform (DCT) is a Fourier transform that can be processed only with a cosine component, which is a real number, because the imaginary term of the Fourier transform becomes zero when the original data are an even function. The discrete Fourier transform is expressed as follows:

(6) $$X\left[k\right]\text{ ​}=\overset{N-1}{\underset{n=0}{\sum }}x\left[n\right]exp\left(-2\pi nkj/N\right),\;\left(k=0,1,･･･,N-1\right)$$

where *x*[*n*] is a discrete signal. If *x*[*n*] is an even function, the imaginary term is zero; therefore, the DCT equation is expressed as follows:

(7) $$X\left[k\right]\text{ ​}=\overset{N-1}{\underset{n=0}{\sum }}x\left[n\right]cos\left(2\pi nk/N\right)\;$$

In this section, the type II DCT of Eqs. (8-1), (8-2), (8-3), which is used in image processing such as JPEG and MPEG, was used for the DCT equation [5, 6, 7].

(8-1) $$X\left[k\right]\text{ ​}=\text{ ​}\sqrt{\left(2/N\right)}\cdot \text{c}\left[k\right]\cdot \overset{N-1}{\underset{n=0}{\sum }}x\left[n\right]･cos\left\{\left(2n+1\right)k\pi /2N\right\}$$

(8-2) $$x\left[n\right]\text{ ​}=\text{ ​}\sqrt{\left(2/N\right)}\cdot \overset{N-1}{\underset{k=0}{\sum }}c\left[k\right]･X\left[k\right]･cos\left\{\left(2n+1\right)k\pi /2N\right\}$$

(8-3) $$\begin{array}{cc} c\left[k\right]\text{ ​}=\text{ ​}1/\sqrt{2},\text{ ​} & k\text{ ​}=\text{ ​}0\\ 1 & k\text{ ​}\neq \text{ ​}0 \end{array}$$

**Fig. 9:**
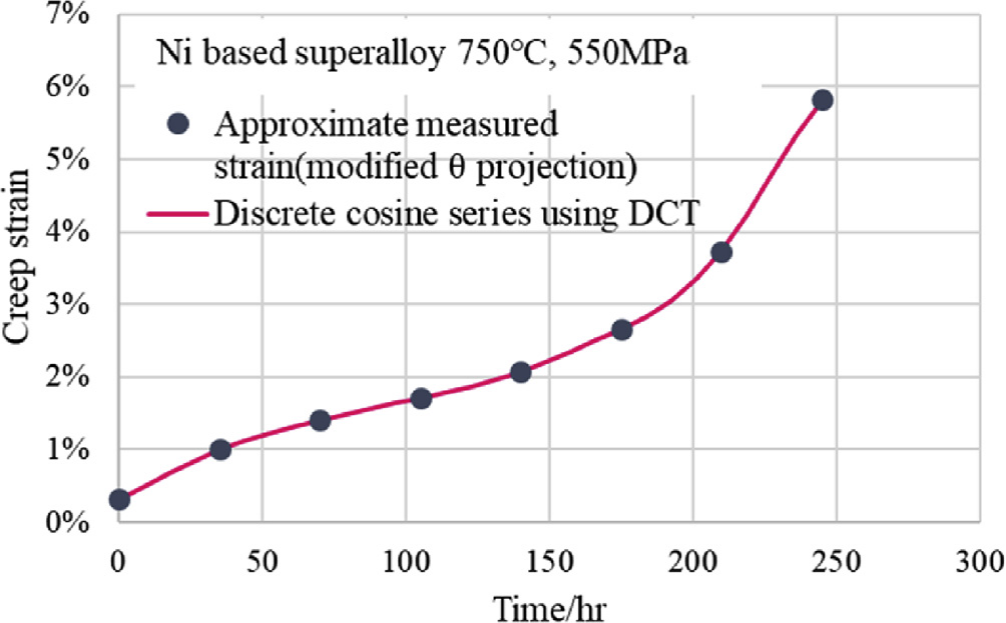
Comparison between calculated curve of discrete cosine series using DCT and measured creep curve.

The creep data used for the discrete cosine transform must be obtained by measuring the creep strain at equal time measurement intervals. However, because the measurement time of the NIMS data was not equally spaced, the measured points were approximated by the modified θ projection, and the equidistant creep strain-time data were calculated from this approximate equation. For example, the creep curve of 750 °C and 550 MPa for the Ni-based alloy of NIMS was approximated by the modified θ projection, and eight points of creep data at 35 h intervals were prepared from the obtained modified θ projection equation. These eight equally spaced data were DCT transformed as x[n] by Eq. (8-1), and a discrete cosine series (inverse discrete cosine transform (IDCT)) was obtained from Eq. (8-2) using the obtained X [k] value (k = 0–7). Although the number of the obtained X[k] is eight, it is very easy to perform the discrete cosine transform needed to obtain X[k] on a computer. Moreover, we can easily use Eq. (8-2) as a creep equation with eight X[k]. Once the eight values obtained for X[k] are input into Eq. (8-2) as coefficients, it is possible to obtain the creep strain using the *n* obtained by dividing the measured time by the interval as a variable. The fitting condition of this discrete cosine series is shown in Fig. 9, where it can be seen that the discrete cosine series was passed through all of the approximated points from the primary creep region to the tertiary creep region. Additionally, the tendency of drawing a sine curve between two points did not occur. This means that if the creep data are measured at equal intervals, it becomes possible to estimate the values in agreement with the measured values by using the discrete cosine series. Moreover, if only eight measured data points are available, estimation by reasonable interpolation becomes possible between two adjacent measured points.

#### 3.4.2 Application of two-dimensional discrete cosine transform to rate constant *α*

The current data of NIMS are not equally spaced for both the stress and temperature. Therefore, based on the NIMS data and the data estimated using the NIMS data, we prepared 8×8 equally spaced data (Fig. 8) and confirmed the effectiveness of the two-dimensional (2D) discrete cosine transform. If this performs as expected, new equally spaced experimental results can be added to future databases to conduct precise analysis using DCT.

In the previous small section, it was found that the one-dimensional discrete cosine transform with time as a variable fit well with the measured value of the creep curve. Therefore, in this small section, a 2D discrete cosine transform with the two variables of temperature and stress was applied to the rate constant *α* shown in Fig. 8. Specifically, the rate constant *α* is expressed by Eq. (9-2), with temperature and stress as the variables. This makes it possible to include the stress variables and temperature variables in the coefficients of the creep equation, and the creep equation of the modified θ projection can have the variables of strain, time, temperature, and stress in their entirety. If the 2D DCT coefficient is *F*[*k*, *l*], the discrete cosine transform and the inverse discrete cosine transform (IDCT) for the 2D discrete signal *f* [*i*, *j*] with a size of N×N are expressed by the following equations [7].

(9-1) $$\text{ ​}F\left[k,l\right]\text{ ​}=\overset{N-1}{\underset{J=0}{\sum }}\text{ ​}\overset{N-1}{\underset{i=0}{\sum }}f\left[i,j\right]\varphi k\left[i\right]\varphi l\left[j\right]$$

(9-2) $$\text{ ​}f\left[i,j\right]\text{ ​}=\text{ ​}\overset{N-1}{\underset{l=0}{\sum }}\;\overset{N-1}{\underset{k=0}{\sum }}F\left[k,l\right]\varphi k\left[i\right]\varphi l\left[j\right]\text{ ​}$$

(9-3) $$\begin{array}{l} \varphi k[i]\;=\;1/\sqrt{N},\;k\;=\;0\\ \;=\;\sqrt{\left(2/\text{N}\right)}\cdot \cos\left\{\left(2\text{i}+1\right)\text{kπ}/2\text{N}\right\},\text{ k }=\;1,2,\cdots \text{N}-1 \end{array}$$

**Fig. 10:**
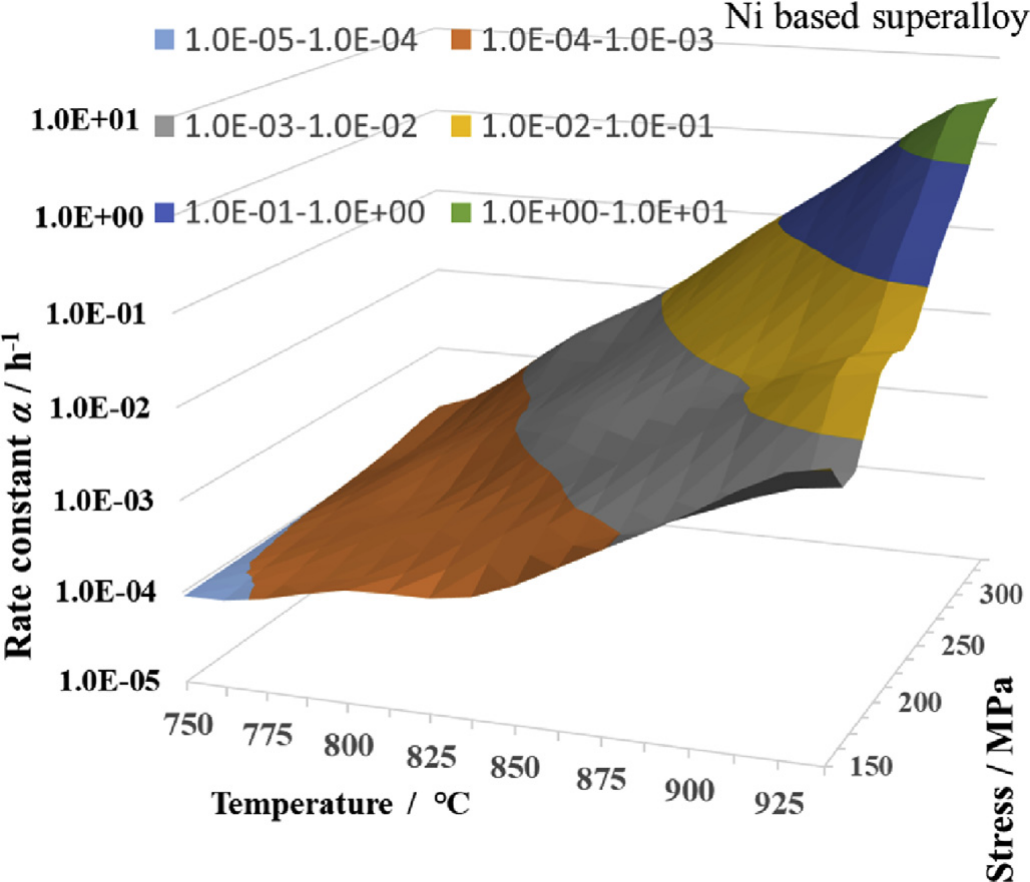
3D contour map of rate constant α calculated by 2 dimensional DCT.

The 2D discrete cosine transform was performed based on the 2D discrete data with a size of 8×8, as shown in Fig. 8, to obtain the 2D discrete cosine transform and series with the two variables of temperature and stress. Thereby, the value of *α*_*ij*_ was calculated and was found to be exactly the same as the value of 64 *α*_*ij*_ (*i* = 0 to 7, j = 0 to 7) shown in Fig. 8. Therefore, the contour map is identical to that shown in Fig. 8. Next, from the 2D discrete cosine series using the previously obtained 8×8 discrete cosine transform coefficient *F*[*k*, *l*], the value of the midpoint between each two adjacent points of the 8×8 2D discrete data was estimated. A contour diagram with a size of 16×16, including these estimated values, is shown in Fig. 10, where reasonable positive values of *α* were observed among the midpoints between two adjacent points of the 8×8 2D discrete data. In Fig. 8, only one point at 937.5 °C and 150 MPa was larger than the surrounding points. However, as is also shown in Fig. 10, the state of Fig. 8 was precisely reproduced. Moreover, the value of *α* tended to increase continuously from 150 MPa to 337.5 MPa at 925 °C, when the slope increased or decreased. Fig. 10 shows the same trend as that shown in Fig. 8. However, at the three points of (912.5 °C, 212.5 MPa), (925 °C, 212.5 MPa), and (937.5 °C, 212.5 MPa), the slope had a small negative value from 200 MPa to 212.5 MPa. This is attributed to the size relationship of the values of the plural points surrounding the three abovementioned points. However, at these three points, *α* had a reasonable positive value. Furthermore, for the original 8×8 data included in the 16×16 data, the same value as that shown in Fig. 8 was obtained. From the above considerations, it was concluded that it is possible to express the rate constant *α* by the two variables of temperature and stress in a single equation.

Here, the vertical axes of Figs. 3, 4, 5, 6, 8, and 9 are simple logarithmic axes. However, *α*_*ij*_ on the vertical axis in Fig. 10 was obtained using the following method. The 2D cosine transform coefficient *F*[*k*, *l*] was obtained by a 2D discrete cosine transform with log_10_*α*_*ij*_ as *f* [*i*, *j*] (Eq. (9-1)). By using this *F*[*k*, *l*], a discrete cosine series *f*[*i*, *j*] was calculated by Eq. (9-2). Then, *α*_*ij*_ was obtained by calculating 10 to the *f*[*i*, *j*]^th^ power. This is the new calculation way how to extinguish some negative values of *α* seen in the author's previous paper [8]. Additionally, when *α*_*ij*_ was displayed on the vertical axis in Fig. 10, the vertical axis was displayed as a common logarithmic axis.

## 4 Conclusion

The following conclusions were drawn from this study.

1) If the calculated value must be consistent with the value at all measured points, and if a waving phenomenon such as a sine curve does not occur, the N-Spline function of Eq. (3) is suitable for the expression of the rate constant *α*, which is included in the modified θ projection (Eq. (1)) for the creep constitutive equation.
2) If the calculated value does not need to coincide completely with the measured value, and an accurate estimation must be made in the region above the middle stress, the modified θ projection type of Eq. (4) is suitable for the expression of the rate constant *α* for the Ni based superalloy. Moreover, the modified θ projection can fit well all of the measurement points including the excluded point, while using all of the measured points except one point at the rightmost high stress. However, in this case, it is necessary to consider the possibility of *α* becoming negative when approaching 0 MPa on the low stress side of the leftmost end point.
3) The power law is evenly close to any measured point. However, we must note the fact that deviating from the measured point of *α* at the maximum stress greatly affects the deviation from the measured point in the tertiary creep region, in comparison with other equations (Fig. 6).
4) The equation of the modified θ projection type fits well not only to the relationship curve between *α* and stress, but also to the relationship curve between *α* and temperature.
5) The one-dimensional discrete cosine series of Eq. (8-2) is suitable to the equation expressing the relationship between the creep strain and time. However, it is required that the creep strain data are measured with equal interval measurement time.
6) The rate constant *α* of the modified θ projection for the creep constitutive equation can be expressed by the 2D discrete cosine series of Eq. (9-2), where the temperature and stress are the two variables. Therefore, to estimate by interpolation, we can obtain the rate constant *α* for each condition of the required temperature and stress by using a single equation. This method simultaneously satisfies the stress dependence and temperature dependence of *α*, while keeping the coefficients of a single equation at fixed values.
7) It was found that the discrete cosine transform and series can be applied not only to the coefficients of the creep equation but also to the creep equation itself. It is very important that the Fourier transform, which is considered to be applicable only to periodic functions, can be applied to non-periodic functions like a creep equation or its coefficients without apodizing such as windowing [9]. Therefore, it has the advantage of saving time and labour to search for the optimal window function.

## Declarations

### Author contribution statement

Hideo Hiraguchi: Conceived and designed the experiments; Performed the experiments; Analyzed and interpreted the data; Contributed reagents, materials, analysis tools or data; Wrote the paper.

### Funding statement

This research did not receive any specific grant from funding agencies in the public, commercial, or not-for-profit sectors.

### Competing interest statement

The authors declare no conflict of interest.

### Additional information

No additional information is available for this paper.
